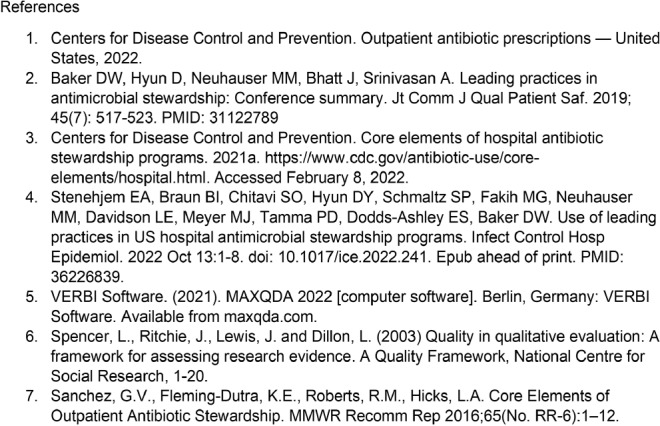# Antimicrobial Stewardship in Hospital Outpatient Clinics: A Qualitative Study of Alignment with Existing Guidance

**DOI:** 10.1017/ash.2025.268

**Published:** 2025-09-24

**Authors:** Salome Chitavi, Michael Kohut, Barbara Braun, Whitney Buckel, Rachel Zetts, David Hyun

**Affiliations:** 1 The Joint Commission; 2 The Joint Commission; 3 Intermountain Healthcare; 4 The Pew Charitable Trusts; 5 The Pew Charitable Trusts

## Abstract

**Background:** It is estimated that 28% of all oral antibiotics prescribed in outpatient care in the United States are inappropriate.1 Most hospitals have established antimicrobial stewardship programs (ASPs) that focus on inpatient antimicrobial stewardship2,3 however, less is known about the engagement of hospital ASPs in outpatient clinics. The goal of this project was to explore the extent to which ASP activities in hospital affiliated clinics align with current guidance for outpatient antibiotic stewardship and challenges to applying hospital based interventions in outpatient settings. **Methods:** The study population comprised 288 hospitals that participated in two previous antimicrobial stewardship studies4. Hospitals needed to have an active outpatient ASP to be included. We conducted in-depth telephone interviews with ASP leaders from 28 diverse hospitals. We used MAXQDA 20225 for data analysis and the framework method6 to organize and analyze interview transcripts based on 4 CDC Core Elements for Outpatient Stewardship.7 **Results:** The sample included 11 large, 9 medium, 8 small hospitals with various outpatient settings (Figure 1). Commitment. Few hospital ASPs had a dedicated outpatient stewardship leader. Around half the hospitals included outpatient sub-committees or representatives on the ASP committee. Only one hospital had a formal stewardship lead at the clinics while others relied on local contacts and EHR system-based interventions. Tracking and reporting. Most hospitals in the sample reviewed outpatient specific data, though many had no data analyst or IT resources for outpatient data. Action. Hospitals in our sample differed widely regarding development of decision-support tools. Only some developed guidelines tailored to outpatient settings, a few of them distributed via CDSS requiring indications for prescriptions, whereas others simply made inpatient guidelines available to clinicians. Education. Only some hospital ASPs developed content tailored for outpatient. Key challenges for expansion of stewardship into outpatient settings included lack of funding, and dedicated staff; lack of clarity regarding best practices for measurement; and an outpatient EHR infrastructure that limited effective guideline distribution and measurement. **Conclusions:** Outpatient stewardship programs were aligned with current guidance, although frequently missing outpatient-specific committee representation, data and education. Few hospitals received additional resources for expansion into outpatient stewardship and most lacked dedicated leaders at clinics, putting additional burden on inpatient ASP leads. While some hospitals have developed guidelines, tracked prescriptions, and provided clinic or clinician feedback, there is need for investment in staff and EHR infrastructure to improve outpatient-specific guideline development, distribution and measurement.

**Figure:**
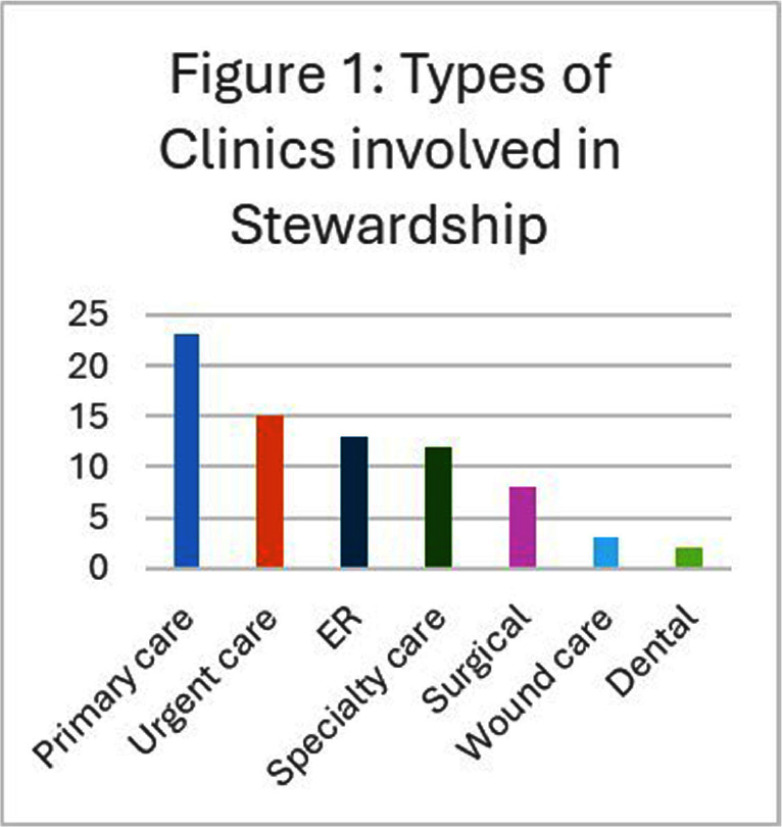

**Figure:**